# Yield optimization, physicochemical characterizations, and antioxidant properties of food grade agar from *Gracilaria tenuistipitata* of Cox's Bazar coast, Bangladesh

**DOI:** 10.1002/fsn3.3265

**Published:** 2023-02-15

**Authors:** Md. Mohibbullah, Md. Abu Talha, Md. Abdul Baten, Asif Wares Newaz, Jae‐Suk Choi

**Affiliations:** ^1^ Department of Fishing and Post Harvest Technology Sher‐e‐Bangla Agricultural University Dhaka Bangladesh; ^2^ Seafood Research Center Silla University Busan South Korea; ^3^ Department of Seafood Science and Technology, The Institute of Marine Industry Gyeongsang National University Tongyeong‐si South Korea

**Keywords:** agar, antioxidant, extraction, *Gracilaria tenuistipitata*, physicochemical properties

## Abstract

The present study was aimed at investigating the optimization of extraction variables for food grade quality agar from *Gracilaria tenuistipitata*, so far, the first study on Bangladeshi seaweed. Water (native)‐ and NaOH (alkali)‐pretreated agars were comparatively analyzed by several physicochemical parameters. All extraction variables significantly affected the agar yield in both extraction conditions. Alkali‐pretreated agar provided a better yield (12–13% w/w) and gel strength (201 g/cm^2^) in extraction conditions as followed by 2% NaOH pretreatment at 30°C for 3 h, seaweed to water ratio at 1:150, and extraction temperature at 100°C for 2 h. Gelling and melting temperatures, color, and pH values of both agars were found to be comparable with commercial agar. Significantly higher sulfate contents including organic and inorganic and total carotenoids were reported in native (3.14% and 1.29 μg/mL) than that in alkali‐pretreated agar (1.27% and 0.62 μg/mL). FTIR spectrum demonstrated the purity of the agar as characterized by the stronger relative intensity with higher degree of conversion of L‐galactose 6‐sulfate to 3,6‐anhydrogalactose in alkali pretreatment group than that of native ones. Moreover, antioxidant activity (% DPPH scavenging) was observed and confirmed by IC_50_ values of 5.42 and 9.02 mg/mL in water‐ and alkali‐pretreated agars, respectively. The results suggested that agar from *G. tenuistipitata* with optimized alkali extraction conditions could promote cost‐effective yield with improved physicochemical characteristics and biofunctional values upon consumption by the consumers as food materials.

## Graphical abstract

Extraction variables affect agar yield in both water‐ and NaOH‐pretreated groups. Among them, 2% NaOH pretreatment of Gracilaria tenuistipitata results cost‐effective agar yield with food grade quality having improved physicochemical characteristics.

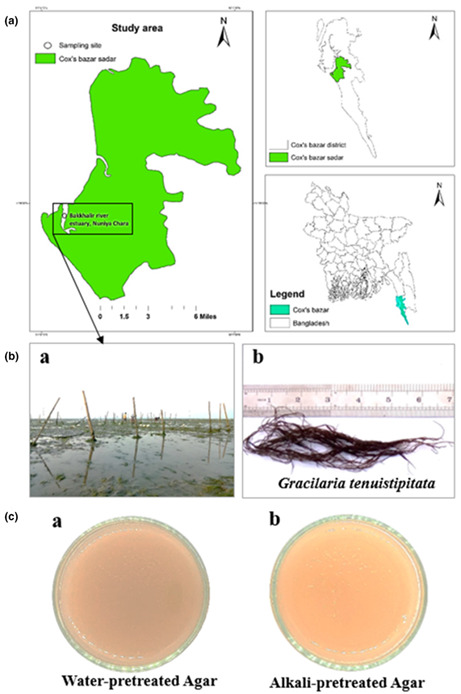

## 1 INTRODUCTION

Agar is a seaweed‐derived gel‐forming substance, also referred to as hydrocolloid or phycocolloid, with a main structural component of polysaccharides, found in the cell walls of particular seaweed species belonging to the class of Rhodophyceae (red algae; Sasuga et al., 2017). Agar is chemically composed of two basic components of agarose and agaropectin. Agarose has gelling properties due to the presence of repeating units of agarobiose with β‐d‐galactopyranosyl and 3,6‐anhydro‐α‐l‐galactopyranosyl groups. Whereas, agaropectin shows the similar structure but with the exception of having 5–10% of sulfate esters, methoxyl groups, and pyruvic acid at different positions in the polysaccharide chains (Sousa et al., 2012; Venugopal, 2016). Agarophyte of red seaweeds has been used as starting materials for commercial agar production worldwide; these species are reported as *Gracilaria* (Gracilariaceae; Gioele et al., 2017), *Gelidium* (Gelidiaceae; Belattmania et al., 2021), *Ahnfeltia* (Phyllophoraceae; Truus et al., 2006), and *Pterocladia* (Gelidiaceae; Brasch et al., 1984). *Gelidium* sp. is well known for its better quality agar, but difficulty in culture limits the use of this species. Among agarophytes, *Gracilaria* is the most contributed species across the world because of its availability in nature and cultivation in many regions and countries (Gioele et al., 2017). The properties of agar determine the aimed uses whether it is applicable to biological works or food industry, since it is largely affected by the species of origin and extraction variables. Keeping in mind, our search has been made to extract agar with the feasibility of commercial uses in the market.

Bangladesh has a 480 km of coastline and 25,000 km^2^ of coastal area with a huge population, supporting a variety of land use practices. Bangladesh host more than 77 genera and approximately 250 seaweed species (Agriculture Organization of the United Nations. Fisheries, D, 2000). *G. tenuistipitata* occurs abundantly in nature and also is being cultivated in Cox's Bazar coast of Bangladesh. However, the post‐harvest utilization of this red seaweed especially agar extraction in a larger scale is not practiced yet, due to the lack of proper knowledge on seaweed handling techniques from raw materials to agar products. Recently, a study performed by (Hossain et al., 2021) reported the agar‐yielding potential of *G. tenuistipitata* collected from Bangladesh coasts. There were no pioneering studies on the optimization of agar extraction conditions and their physicochemical properties with health functional characterizations, which is essential to be employed in food applications. Although the extraction methodology of *Gracilaria* sp. is well studied in seaweed‐cultivated countries, however, agar yield and quality differ significantly based on geographic locations from where it grows.

Agar is being utilized in the food industry as texture modifying and thickening agents to prepare other desired foods and also in the biological field, especially microbiology. Despite these uses, an alternative approach is currently being employed on biodegradable films (Guerrero et al., 2014), encapsulation technology (Alehosseini et al., 2018), biopolymeric nanofibers (Sousa et al., 2015), and health functionalities such as antioxidant and antimicrobial activities upon consumption (Chen et al., 2005). Agar yield of *Gracilaria sp*. decreases with the increase in NaOH concentration, and it varies up to 10–50% depending on the species and season. The extraction time and temperature have greatly influenced the yield, gel strength, and quality characteristics of agar produced from *Gracilaria sp*. (Martínez‐Sanz et al., 2019). Since alkali pretreatment is critical for obtaining quality agar, alternatively, it dramatically reduces the extraction yield (Meena et al., 2008). To prevent this yield degradation, alkali concentrations and extraction variables should be optimized before considering commercial applications. Together with alkali pretreatment, a simple and conventional method of native agar extraction needs to be investigated to compare the feasibility and sustainability in applications of food industry. Elimination of alkali pretreatment step may cause nonpurified agar containing many compounds like proteins, polyphenols, and pigments like carotenoids, those might have antioxidant properties (Martínez‐Sanz et al., 2019). Therefore, optimization of agar yield, physicochemical properties, structural characterization, and antioxidant activities were investigated in water and alkali pretreatment groups, considering cost‐effective to the target applications.

## 2 MATERIALS AND METHODS

### 2.1 Chemicals

All chemicals used in this study were analytical grade and purchased from Sigma Chemical Company, unless otherwise stated.

### 2.2 Collection, identification, and preparation of *Gracilaria tenuistipitata*

**FIGURE 1:**
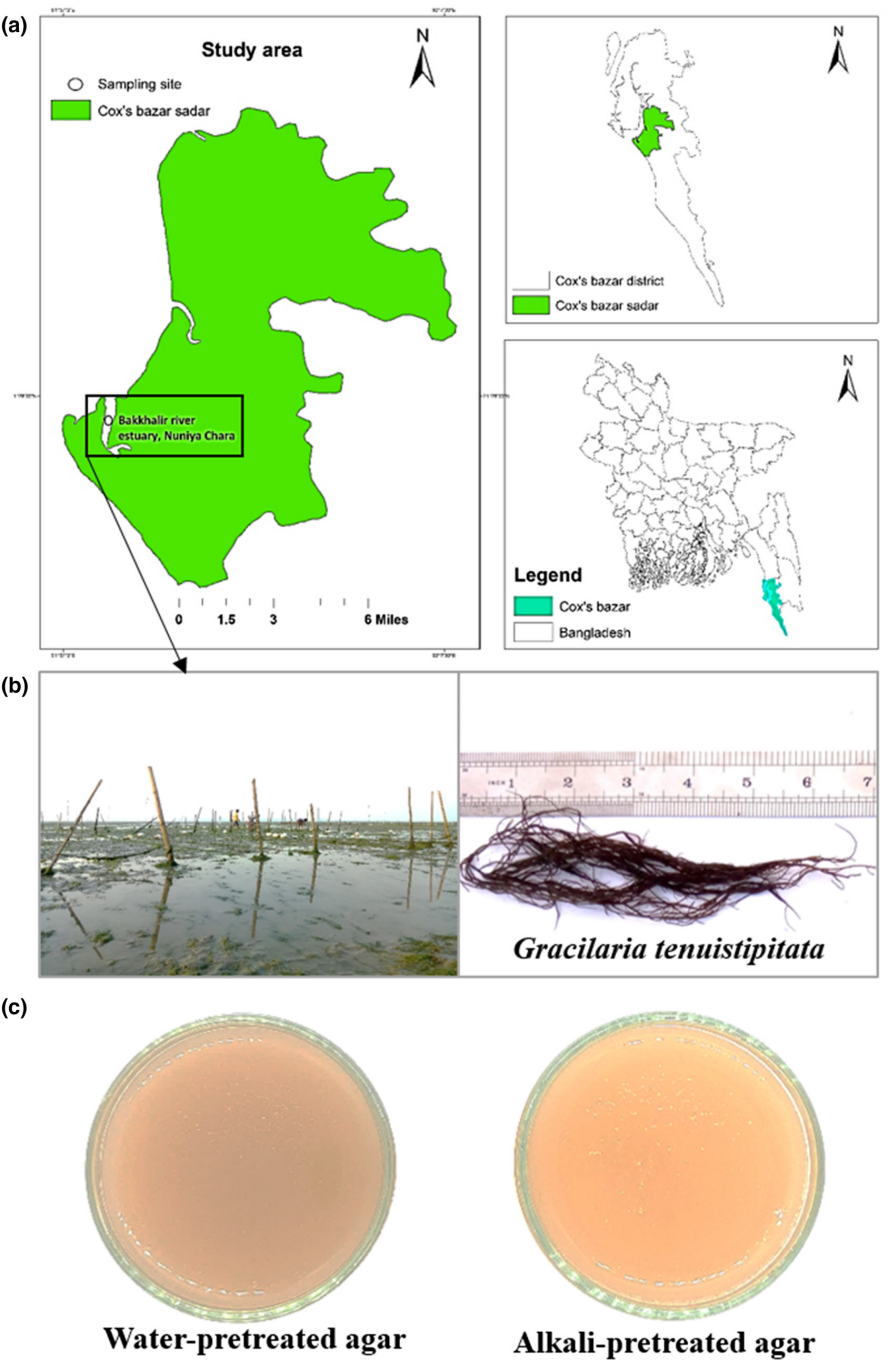
Seaweed collection site, Cox's bazar, Bangladesh (a) culture plot at Nuniarchara area and harvested seaweed of *Gracilaria tenuistipitata* (b) and water‐ and alkali‐pretreated agars extracted from *Gracilaria tenuistipitata* (c).

Seaweed was collected from the shore and subtidal region of Nuniarchara sea beach, Cox's Bazar (21°28′27″ N and 91°57′52″ E) in March 2021 (Figure 1A,B). The collected seaweed was transported in a large plastic container with adequate seawater to the Fishing and Post Harvest Technology Department at Sher‐e‐Bangla Agricultural University, Dhaka, Bangladesh. The samples were properly washed with running water and drained off the sand and other dirt materials. The clean sample was in shade drying at RT for 3–5 days, packed in plastic zipper bags, and placed in dark condition until use.

### 2.3 Water and alkaline pretreatment for agar extraction

#### 2.3.1 Soaking time

A total of 12 samples each consisting of 5 g (dry basis) clean seaweed were taken and, among them, nine samples were soaked for 1, 2, and 3 h at room temperature (25°C) to hydrate. The left three samples were used as control and were soaked (0 h). All samples were then boiled for 2.5 h in 250 mL conical flasks with distilled water at 90°C in a water bath for extraction.

#### 2.3.2 Soaking temperature

All the boiled sample was soaked at 30, 35, and 40°C for 2 h. On the contrary, the remaining three samples were soaked at RT (25°C) and used as a control. Extraction was carried out following the same procedure as mentioned before.

#### 2.3.3 Seaweed to water ratio

Samples from the previously prepared groups were soaked for 2 h at 30°C in different volumes of water to represent variable seaweed–water ratios. Five different seaweed–water ratios were (1:25, 1:50, 1:100, 1:150, and 1:200) used and transferred to a water bath for agar extraction, as followed by the same procedure.

#### 2.3.4 Extraction temperature

Optimized seaweed–water ratio of 1:150 was used to know the extraction temperature. For this purpose, extraction was carried out at four different temperatures of 70, 80, 90, and 100°C by boiling the samples for 2.5 h in a water bath (TWB‐22D, Taisite Lab Sciences Inc.).

#### 2.3.5 Extraction time

Extraction from the samples was carried out at 100°C at five different times of 1.0, 1.5, 2.0, 2.5, and 3.0 h in a water bath.

### 2.4 Evaluation of agar yield

Then, extracted samples were filtered using a three‐ply cheesecloth and transferred to plastic containers (500 mL). The filtrate was frozen overnight, thawed at RT for 4 h, and then freeze‐dried at −110°C (VC‐2200, LABOGENE, GYROZEN Co. Ltd.) until complete dry. The quantity of agar was determined in terms of agar yield expressed as a percentage on a dry basis and calculated from the following equation:

$$\begin{array}{c} \%\text{Agar Yield}=\left[\left(\mathrm{Dry}\;\text{weight of agar}\;\left(g\right)/\mathrm{Dry}\;\text{weight of seaweed}\;\left(g\right)\right)\right]\\ \;\times 100 \end{array}$$

### 2.5 Alkali preparation and treatment

Five different concentrations of 1%, 2%, 3%, 4%, and 5% alkali solutions were prepared by dissolving analytical grade sodium hydroxide in distilled water. The dried samples (5 g) were soaked in each alkali concentration of 1%, 2%, 3%, 4%, and 5% for 3 h at room temperature in 250 mL conical flask. The flasks were then placed for 1 h in a water bath at 70, 80, 90, and 100°C, respectively. After alkali treatment, the samples were washed with running tap water for 1 h to remove excess alkali. Extraction was carried out by boiling the sample for 2.5 h in 250 mL of distilled water at pH 7.0–7.5. The extracts were filtered using three‐ply cheesecloth and transferred to plastic containers (500 mL).

### 2.6 Physicochemical properties of agar gels

Agar gels were prepared according to the method of Kumar and Fotedar (2009). Agars were dissolved in boiling DW on a hot plate stirrer to keep a final concentration of 1.5% (w/v) and stirred until complete solubilization. The boiled agar was poured onto Petri dishes with 5 cm diameter and 1 cm height and incubated at refrigerated temperature (4°C) for 12 h and then equilibrated at RT for 3 h before analysis. A commercial agar was used as a reference control.

#### 2.6.1 Gel strength

The gel strength of agar was performed using a texture analyzer (Brookfield Texture Analyzer, Massachusetts, USA), with a 12.5 mm diameter of the cylindrical probe and a load cell of 7 kg, at a penetration rate of 1 mm s^−1^, as per the modified method of Lee et al. (2014). The maximum force (g) was recorded when plunger penetrated 5 mm into the agar gels, and the result was expressed as g/cm^2^.

#### 2.6.2 Melting and gelling temperatures

Melting and gelling temperatures were determined using techniques described by Freile‐Pelegrın and Murano (2005) with minor modifications. Melting point of the gel in test tubes was measured by placing a glass bead (3 mm diameter) on the gel surface. The test tube rack with test tube was transferred to the water bath at boiling temperature. The melting point was recorded with a digital thermometer when the glass bead sank into the solution. Same test tubes were kept at room temperature to measure the gelling temperature. The tubes were tilted up and down in a water bath at room temperature until the glass bead ceased moving. The gel temperature in the test tube was immediately measured by introducing a digital thermometer into the agar gel.

#### 2.6.3 Color characteristics

The color of agar gels was measured using a colorimeter (CM‐700d Spectrophotometer; Konica Minolta Sensing Inc.) and reported in CIE system, where hunter system values including L* (lightness), a* (redness), and b* (yellowness) were monitored.

#### 2.6.4 Agar pH

The pH meter (ST3100, Ohaus) was used to assess the agar pH. A 5 g of the sample was added to 25 mL of DW. Each sample was independently measured three times and expressed as an average pH value.

### 2.7 Chemical compositions of agar

#### 2.7.1 Sulfate content analysis

The sulfate content of agar samples was evaluated using a spectrophotometric microplate reader, based on BaCl_2_‐gelatin method (Torres et al., 2021). Barium chloride‐gelatin reagent was prepared with 75 mg gelatin powder with 25 mL of ultrapure water in screw cap tube, incubated at 80°C for 10 min, and homogenized the solution completely using a vortex. After that, 250 mg BaCl_2_ was added to the gelatinous solution under stirring condition and the reagent was kept at 4°C up to 7 days for further use. Preparation of hydrolyzed sample was made by mixing 1 mg dry agar with 250 μL of 0.5 mol/L HCl, vortexing for 60 s, incubating in a drying oven at 105°C for 3 h, and finally centrifugation at 13, 400 *g* for 15 min at RT. The collected supernatant was used to assess the organic sulfate content in agar. The nonhydrolyzed sample was prepared by adding 5 mg dry agar with 500 μL ultrapure water. The 100 μL of supernatant after centrifugation using the aforementioned condition was mixed with 125 μL of 1 mol/L HCL and 25 μL ultrapure water, placed into an ice bath, and performed just before microplate reading (SPECTROstar Nano, BMG LABTECH). The first reading was made as a reference point on a solution containing a 20 μL sample (hydrolyzed and nonhydrolyzed) and 140 μL of 0.5 mol/L HCL, using a microplate reader at the absorbance value of 405 nm. The second reading was taken after mixing 40 μL BaCl_2_‐gelatin reagent into each well. The total sulfate content was expressed as a percentage of dry weight using the standard calibration equation of sulfate ions (1.95–500 μg/mL) as follows:

$$y=0.0034^{*}\;X+0.1191;R^{2}=0.9643$$

#### 2.7.2 Fourier transform infrared (FTIR) spectroscopy

Fourier transform infrared analysis of two different agars was performed to determine the characteristic peaks spectroscopically with their functional groups using a NICOLET model iS50 FTIR (Thermo Fisher Scientific), using a diamond single reflection attenuated total reflectance, iS50 ATR (Thermo Fisher Scientific). The spectra appeared in the wavelength of 4000–400 cm^−1^. The spectral data were collected and processed using OMNIC 9.0 Software (Thermo Scientific).

#### 2.7.3 Determination of chlorophylls and total carotenoids

The chlorophyll a, chlorophyll b, and total carotenoids were determined spectrophotometrically, following the procedure of (Hossain et al., 2021). With minor modifications, 10 mg dried agar of each group was mixed with 1 mL acetone‐water (4:1) solvent, sonicated for 3 min (5 cycles: pulse 30 sec, pause 10 s), and centrifuged at 5000 rpm for 10 min at 20°C. The absorbance maxima for each supernatant were read at 663, 646, and 470 nm and calculated from the equations below:

$$\text{Chlorophyll}\;a\;\left(\mathrm{\mu g}/\mathrm{mL}\right)=12.25\;A_{663}–2.25\;A_{646}$$

$$\text{Chlorophyll}\;b\;\left(\mathrm{\mu g}/\mathrm{mL}\right)=20.31\;A_{646}–4.91\;A_{663}$$

$$\begin{array}{c} \text{Total carotenoids}\;\left(\mathrm{\mu g}/\mathrm{mL}\right)\\ =\left[1000\;A_{470-}2.27\;\left(\mathrm{Chl}\;a\right)–81.4\;\left(\mathrm{Chl}\;b\right)\right]/227 \end{array}$$

### 2.8 Antioxidant activity by DPPH (2, 2‐diphenyl‐1‐picrylhydrazyl) scavenging assay

The free radicals of DPPH scavenged by agar extracts of three different concentrations (1, 5 and 10 mg/mL) were carried out with negligible modifications from the previous study. A 40 μL of sample or standard was dissolved in 260 μL 0.1 mM methanolic DPPH and incubated for 30 min in the dark at RT. The absorbance was read at 517 nm wavelength using a microplate spectrophotometer. Ascorbic acid (7.3 to 62.5 μg/mL) of the aqueous solution was used as positive control. Different concentrations were employed to calculate IC_50_ value of each sample, indicating that the amount of antioxidant needed to initially decrease the DPPH ions by 50%. The percent of free radical scavenging activity of the agar extracts was calculated using the following equation:

$$\begin{array}{c} \text{DPPH scavenging activity}\;\left(\%\right)\\ =\left[\left(\text{Absorbance of control}–\text{Absorbance of sample}\right)/\text{Absorbance of control}\right]\\ \times 100 \end{array}$$

### 2.9 Data analysis

Data were presented as mean ± SE (*n* = 3). Statistical comparisons will be made by one‐way analysis of variance (ANOVA) with post hoc Duncan multiple comparisons (SPSS software, version 16.0). Predetermined *p* values ≤.05 will be considered statistically significant.

## 3 RESULTS AND DISCUSSION

### 3.1 Optimization of alkali concentration for agar yield

**FIGURE 2:**
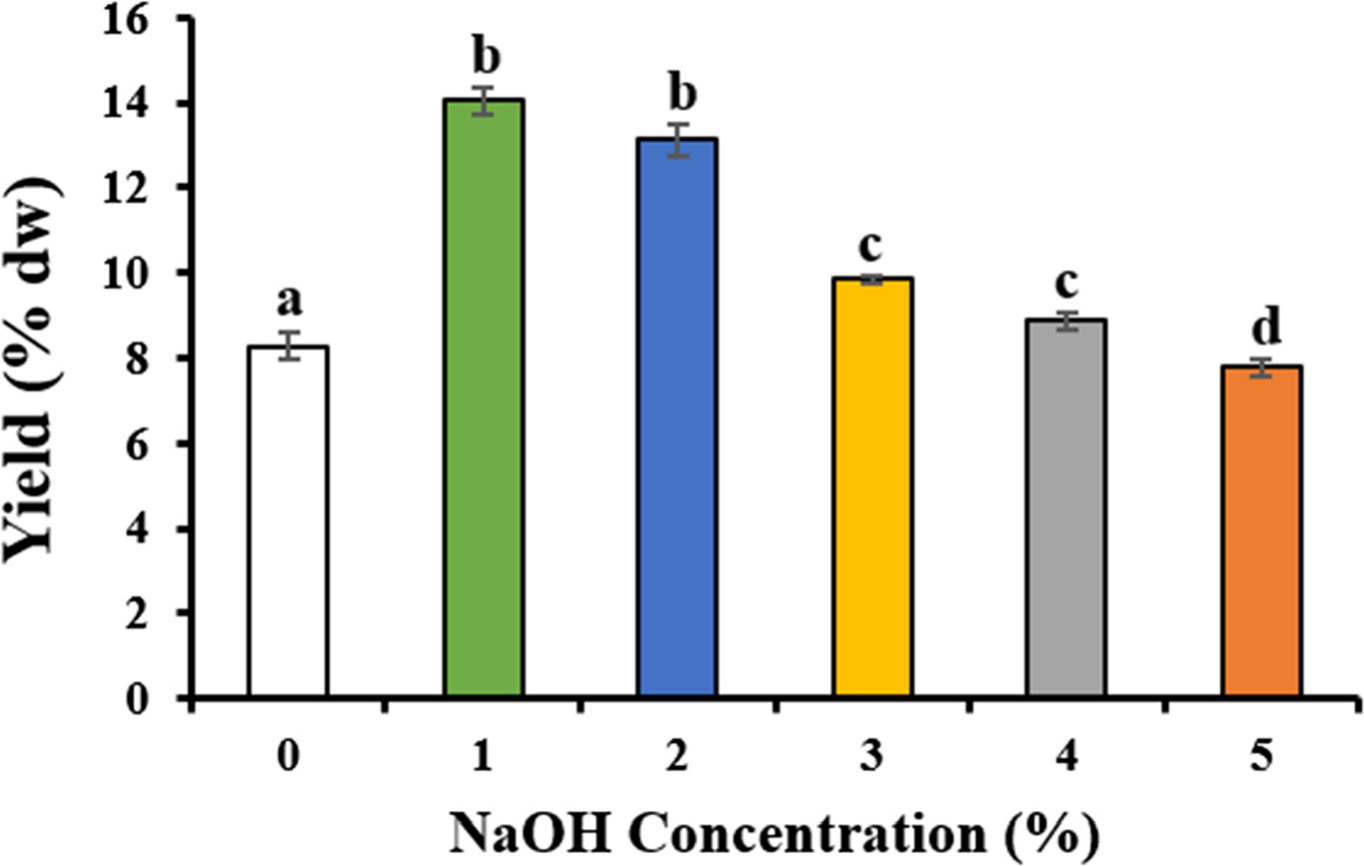
Optimization of NaOH (alkali) concentration for pretreatment of *Gracilaria tenuistipitata* for agar yield in dry weight. Data were presented as mean ± SE (*n* = 3), different letters in superscript showing the statistically significant at 95% confidence level (*p < .05).

The study found that different concentrations of NaOH pretreatment at 0%, 1%, 2%, 3%, 4%, and 5% (w/v) were applied to obtain agar yields of 8.27%, 14.05%, 13.12%, 9.84%, 8.86%, 7.78% and (w/w), respectively (Figure 2). Agar yields of *G. tenuistipitata* treated with 1% and 2% alkali were remained non‐significant difference but significant (*p < .05*) against 0% alkali group (no pretreatment). Agar content was decreased with the increase in NaOH concentrations when investigated on *Gracilaria cervicornis*, *Gracilaria blodgettii*, and *Gracilaria crassissima* species, growing on Yucatán peninsula (Freile‐Pelegrın & Murano, 2005). Alkali treatment from 3 to 5% significantly decreased the agar contents, which were below 10%. A recent study showed maximum agar yield pretreated with 6% NaOH was less than 10%, but authors only used 6% and 8% of NaOH (Hossain et al., 2021). Moreover, another opposite findings by Kumar and Fotedar (2009), a significantly lower (*p < .05*) agar yield was reported in case of 2% NaOH concentration, whereas a significantly higher yield was mentioned in 1% and 3% NaOH concentration for *Gracilaria cliftonii*. However, the percentage of yield reported in the present study is lower than that of reported by Kumar and Fotedar (2009) (*G. cliftonii*, 22% db); Durairatnam (1987) (*G. edulis*, 21.8% db), and Hurtado‐Ponce and Umezaki (1988) (*G. verrucosa*, 21.1% db). This difference in agar yield might be due to the differences in extraction variables like alkali concentration, temperature, and time for the extraction process (Arvizu‐Higuera et al., 2008). However, excessive alkali concentration improves agar quality but decreased agar yield with the loss of many other biologically important compounds. However, the low gelation hysteresis occurred when 0.5 and 1% NaOH pretreatment was applied in *G. cornea*, resulting from interference of charged groups of sulfate ions with intermolecular hydrogen bonds in the aqueous agar medium. Therefore, considering yield and quality of agar, 2% NaOH pretreatment for extraction of *G. tenuistipitata* agar was selected in the present study, which was higher than those evident by the required yield of industrial applications (>8%; Armisen, 1995).

### 3.2 Optimization of agar extraction conditions in water and alkali pretreatment groups

Optimizing the agar extraction process from each *Gracilaria* species is an important step to maximize agar yield with improved agar quality, especially when it is considered for commercial application (Yousefi et al., 2013). The soaking time and temperature affect the accessibility of agar as a base‐soluble polysaccharide of seaweed, which readily interacts with water molecules via hydrogen bonding. The study found different soaking time (0, 0.5, 1, 2 and 3 h) influences on the agar yield in both water and alkali pretreatment groups. The significantly (*p* < .05) higher agar yield in water and alkali treatment obtained at soaking time of 1 and 3 h 21.81 ± 1.02% and 12.43 ± 0.29%, respectively (Table 1). The current finding is quite similar to the values reported by Kumar and Fotedar (2009), where the authors noted the highest agar yield (61.2%) for 1 and 2 h of soaking time. By contrast, Yousefi et al. (2013) reported the highest agar yield in case of 0 h of soaking time. Similarly, soaking temperature influenced the agar yield in water and alkali pretreatment. The significantly (*p* < .05) higher agar yield (21.54 ± 0.31% and 12.10 ± 0.28%) was observed at 35°C and 30°C soaking temperature for water and alkali treatment groups, respectively (Table 1). The results reiterated that the extended soaking time and temperature might be the cause of higher diffusion into the water causing lower agar yield.

**TABLE 1:** Agar yield (% dw) of *Gracilaria tenuistipitata* to optimize the extraction conditions in water‐ and alkali‐pretreated groups.

	Water‐extracted agar yield (% DW)					Alkali‐extracted agar yield (% DW)				
**Soaking time**	**0 h**	**0.5 h**	**1 h**	**2 h**	**3 h**	**0 h**	**1 h**	**2 h**	**3 h**	**4 h**
	11.76 ± 0.24^a^	14.27 ± 0.41^b^	21.81 ± 1.02^c^	21.72 ± 0.32^c^	19.83 ± 0.36^d^	9.3 ± 0.29^a^	9.95 ± 0.29^b^	10.49 ± 0.11^c^	12.43 ± 0.29^c^	11.46 ± 0.29^d^
**Soaking temperature**	**25°C**	**30°C**	**35°C**	**40°C**		**25°C**	**30°C**	**35°C**	**40°C**	
	13.01 ± 0.24^a^	15.35 ± 0.31^b^	21.54 ± 0.31^c^	12.12 ± 0.16^d^		9.84 ± 0.22^a^	12.10 ± 0.28^b^	10.70 ± 0.32^a^	9.95 ± 0.22^a^	
**Seaweed to water ratio**	**1:50**	**1:100**	**1:150**	**1:200**		**1:50**	**1:100**	**1:150**	**1:200**	
	15.71 ± 0.24^a^	17.68 ± 0.18^b^	19.74 ± 0.32^c^	16.60 ± 0.24^d^		8.22 ± 0.11^a^	11.14 ± 0.29^b^	12.54 ± 0.29^c^	11.24 ± 0.11^b^	
**Extraction temperature**	**70°C**	**80°C**	**90°C**	**100°C**		**70°C**	**80°C**	**90°C**	**100°C**	
	11.13 ± 0.47^a^	14.72 ± 0.47^b^	17.33 ± 0.88^b^	20.00 ± 1.53^bc^		9.30 ± 0.11^a^	10.05 ± 0.19^b^	11.24 ± 0.11^c^	12.00 ± 0.19^d^	
**Extraction time**	**1 h**	**1.5 h**	**2 h**	**2.5 h**	**3 h**	**1 h**	**1.5 h**	**2 h**	**2.5 h**	**3 h**
	15.33 ± 0.33^a^	16.00 ± 0.58^ab^	17.33 ± 0.58^b^	21.00 ± 0.58^c^	16.33 ± 0.33^ab^	9.62 ± 0.11^a^	10.38 ± 0.19^b^	12.65 ± 0.32^c^	11.46 ± 0.11^d^	10.92 ± 0.22^bd^

Data were presented as mean ± std (*n* = 3), different letters in superscript showing the statistically significant at 95% confidence level (*p* < .05).

Seaweed–water ratios have positive effects on the agar yield. The significantly (*p* < .05) higher agar yield found at 1:150 seaweed–water ratio in both water and alkali treatments, respectively (Table 1). This finding is in line with the observation of Kumar and Fotedar (2009) with maximum yield at 1:150 seaweed–water ratio, but opposite to those mentioned by Yousefi et al. (2013) with 1:100 seaweed–water ratio. The different extraction temperatures significantly influenced the agar yield of *G. tenuistipitata*. The study found significantly (*p* < .05) higher agar yield at 100°C extraction temperature for both water and alkali pretreatment groups, 20.00 ± 1.53% and 12.00 ± 0.19%, respectively (Table 1). This finding is similar to the previous study on *G. cliftonii*, which yielded maximum agar of 60.6% at extraction temperature of 100°C (Kumar & Fotedar, 2009). Extraction temperature, however, is contrary to the findings of (Wang et al., 2017) with *G. tenuistipitata* at 80°C and Yousefi et al. (2013) with *G. corticata* at 60°C for elevated agar yield. These dissimilarities of extraction temperature vary with different species and might be corroborated with species specific.

The study is evident that extraction time was one of the most important variables in the extraction process as agar yield showed significant differences at different extraction times. The study found significantly higher (*p < .05*) agar yield at 2.5 and 2 h extraction time in both water and alkali pretreatment groups, 21.00 ± 0.58% and 12.54 ± 0.43%, respectively (Table 1). On the contrary, a scholar found higher agar yield in 1.5 h extraction time with 6% alkali treatment (Wang et al., 2017). The present results are quite similar to the findings of Kumar and Fotedar (2009), where the author reported similar range of extraction time to obtain the highest agar yield. The current result of declining agar yield by extending the extraction time may be the cause of degradation and diffusion of agar in aqueous medium.

### 3.3 Physicochemical characteristics of agar gel for both water and alkali pretreatment

#### 3.3.1 Gel strength

**FIGURE 3:**
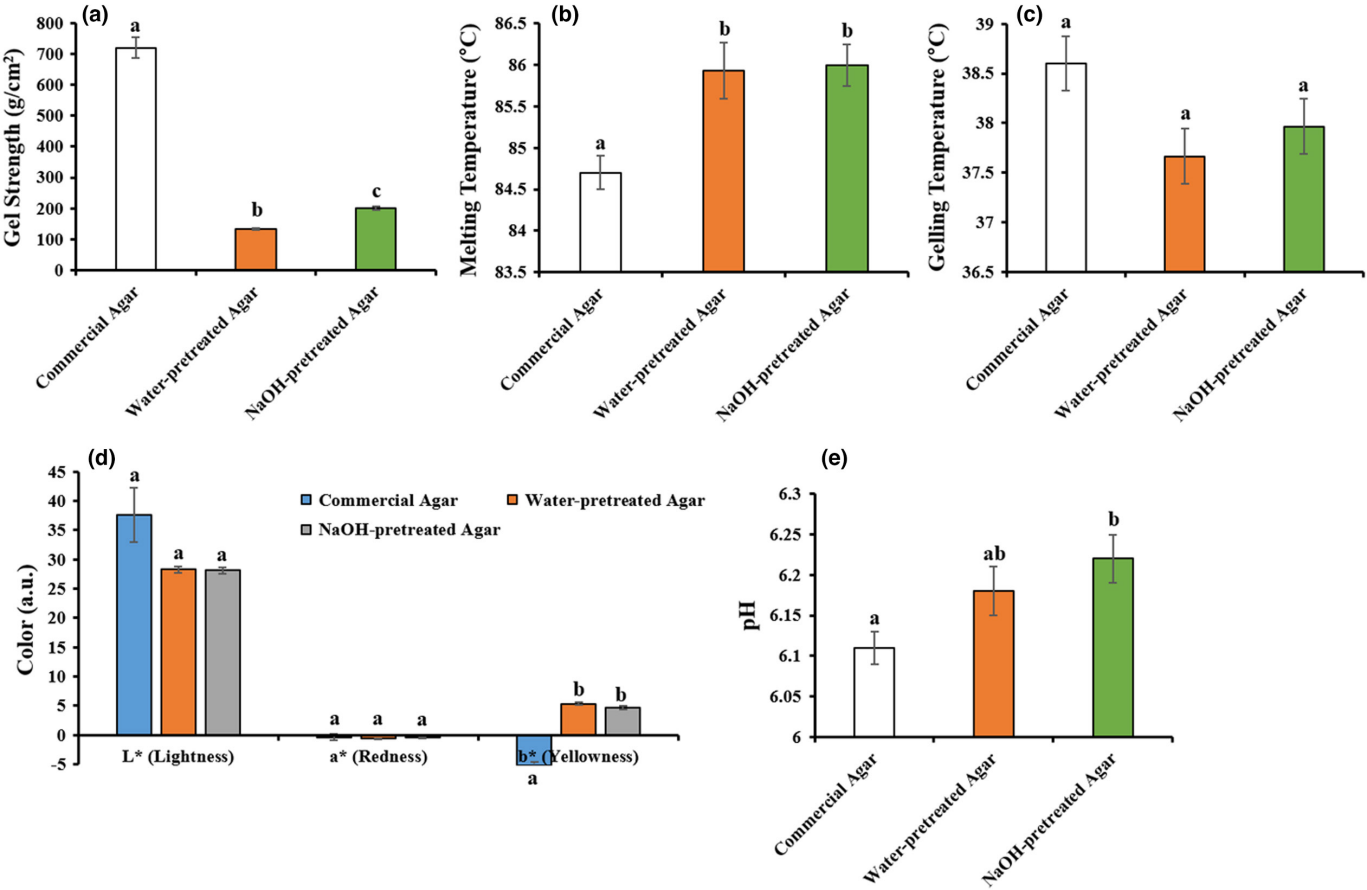
Physicochemical properties of gel strength (a), melting (b) and gelling (c) temperatures, coloration (d) and pH value (e) in water‐ and NaOH‐pretreated agars. Data were presented as mean ± SE (*n* = 3), different letters in superscript showing the statistically significant at 95% confidence level (*p < .05).

Gel strength is often used as a reference parameter for determining the agar quality. For microbiological applications, high‐quality agars with gel strengths >700 g/cm^2^ (in 1.5% w/w solution) are often required on the international market, while agars with lower gel strength values (30–200 g/cm^2^) could be preferred for food industry applications (Armisen, 1995). Gel strengths of water‐ and NaOH‐pretreated agar were recorded as 132.78 ± 2.99 g/cm^2^ and 201.33 ± 5.44 g/cm^2^ (Figure 3a), respectively, and the value noted in NaOH‐pretreated agar was significantly higher (*p < .05*), when compared to water‐pretreated agar. This study was in line with the study of same species but in different geographic locations, where lowest gel strength observed in native agar (without treatment) and increased gel strength while using alkaline pretreatment (Yarnpakdee et al., 2015). It is evident that agar gel strength increased in NaOH pretreatment of *Gelidium* sp. and *Pyropia yezoensis* (Sasuga et al., 2017). The previous studies were in accord with the present findings as gel strength 138 g/cm^2^ and 459 g/cm^2^ have been reported for the agars extracted from *Gracilaria cliftonii* (Kumar & Fotedar, 2009) and *Gracilaria corticata* (Yousefi et al., 2013), respectively. It is known that alkali pretreatment converts L‐galactose‐6‐sulfate into 3,6‐anhydro‐L‐galactose, thereby improving the gel‐forming ability of agar and resulting in increased gel strength (Freile‐Pelegrın & Murano, 2005; Yousefi et al., 2013). Based on the results, our study suggested that alkali‐pretreated agar was much acceptable over native agar (water‐pretreated) for food applications commercially.

#### 3.3.2 Melting temperature

The melting temperature of water‐ and NaOH‐pretreated agar was 85.93 ± 0.34°C and 86.00 ± 0.25°C, respectively, indicating no significant difference (Figure 3b). Our study is very much evident with the previous study of Kumar and Fotedar (2009), as observed 83.0–86.0°C melting temperature of agar produced from *Gracilaria cliftonii*. Although similar melting temperature observed in native agar (85.6°C), higher melting temperature (102.9–103.5°C) reported in alkali‐pretreated agar of *G. tenuistipitata* (Yarnpakdee et al., 2015). Another study found slightly higher melting temperature (88.74–93.86°C) observed in *Gelidium latifolium* for the production of agar (Öğretmen & Kaya, 2019). Melting temperature was significantly higher in water‐ and NaOH‐pretreated agar, as compared to commercial agar, due to different chemical compositions resulting from different extraction materials and processes. In addition, melting temperature of agar gel is positively correlated with its molecular weight; however, we did not analyze the molecular weight of agar in the present study.

#### 3.3.3 Gelling temperature

Gelling temperatures of commercial, water‐, and NaOH‐pretreated agar were recorded as 38.6 ± 0.15°C, 37.67 ± 0.22°C, and 37.97 ± 0.12°C, respectively, indicating no significant differences and slightly lower than commercial agar (Figure 3c). The lower gelling temperature might be due mainly decreased amount of methoxyl group in the agar polymer structure (Xiao et al., 2021). However, the study is similar to the findings noted by Zhao et al. (2020) (36.10 ± 0.22 for dry *Gracilaria tenuistipitata*), Prasad et al. (2007) (36 ± 0.65–42 ± 0.95°C for *Gelidiella acerosa*), and Freile‐Pelegrin et al. (1995) (35.7–39.3°C for *Gelidium canariensis*) and slightly higher gelling temperature observed in *Gelidiella* sp. (42–45°C) and *Gracilaria* sp. (40–42°C), respectively, (Armisen, 1995). Thus, it can be said, seaweeds producing agar with lower gelling temperature are desired to make commercial agar as it prevents heat damage to the materials (e.g., antibiotics) added into hot agar solution (McHugh, 2003). To support the present result, there were no significant differences in gelling temperature of agar between *Gelidium sp*. and *Pyropia yezoensis* when pretreated with NaOH (Sasuga et al., 2017).

#### 3.3.4 Color value

The color is an important attribute, which directly reflects consumer choice. The instrumental color values are depicted as L* for lightness (lightness/darkness: higher/lower), a* for red/green (higher/lower), and b* for yellow/blue. The lightness and redness of water‐ and NaOH‐pretreated agars were nonsignificant but remarkable difference against commercial agar, but yellowness of commercial agar significantly (*p* < .05) varied with water‐ and NaOH‐pretreated agar (Figure 3d). These variations of color within groups were significantly affected by the alkali concentrations. This study was in agreement with Yarnpakdee et al. (2015) observed differences in color of agars made from *G. tenuistipitata* with alkaline (NaOH and KOH) or without treatment at various concentrations. The pigment of agar was removed from alkaline pretreatment of *Gracilaria lemaneiformis* (Li et al., 2008). Moreover, water‐pretreated native agar showed a dark brown appearance that might negatively affect the consumer attraction when use in foods (Figure 1C). This coloration of nonpurified agar without alkali pretreatment was most likely due to the cause of dispersed some proteins, pigments, and polyphenols in hot water solution while extraction (Figure 1C; Martínez‐Sanz et al., 2019). Based on coloration, alkali‐pretreated agar extracted from *G. tenuistipitata* was much acceptable for food applications than those of water‐pretreated native agar.

#### 3.3.5 pH value

The pH value of water‐ and NaOH‐pretreated agar was noted as 6.18 ± 0.03 and 6.22 ± 0.03, which is slightly higher than commercial agar 6.11 ± 0.02 (Figure 3e). According to Food and Agricultural Organization, agar can be used in a wide range of pH from 5 to 8 (Joint & World Health Organization, 2007). A study by Istini et al. (1994) reported that *Gracilaria* sp. agar gel pH range 6.52–6.75. Another study conducted on pH values of extracted agar from *G. latifolium* varied in different season and were range between 6.35 and 7.40 (Öğretmen & Kaya, 2019). Moreover, the pH values between 6.5 and 8 from *Gracilaria tenuistipitata* (Israel et al., 1999) have been reported. Comparing with the present study, the pH values of extracted agar from water‐ and alkali‐pretreated groups were slightly lower because of different harvesting location and extraction condition.

### 3.4 Chemical properties of water‐ and NaOH‐pretreated agar

#### 3.4.1 Sulfate content

**FIGURE 4:**
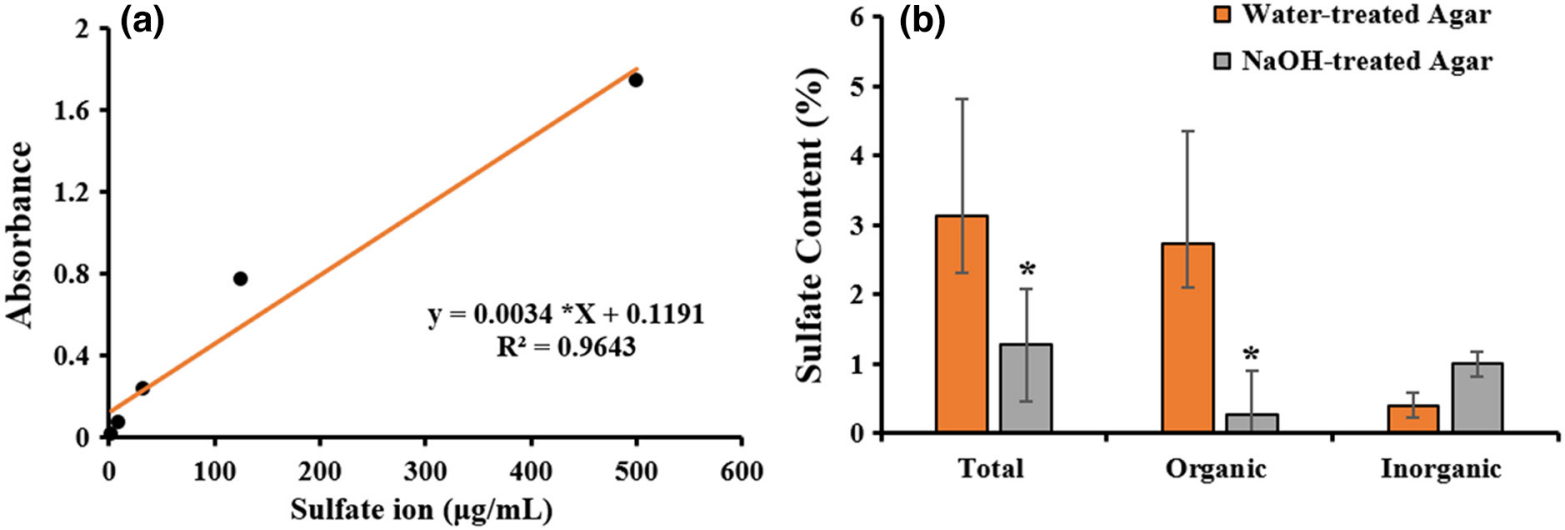
Sulfate contents in water‐ and NaOH‐pretreated agars. Standard calibration equation of sulfate ions for quantifying sulfate contents (a), total, organic, and inorganic sulfate contents in water‐ and NaOH‐pretreated agars (b). Data were presented as mean ± SE (*n* = 3), statistically significant at 95% confidence level (*p < .05).

Many biological activities of sulfated polysaccharides are positively related to the presence of sulfate groups either in free or esterified forms (Torres et al., 2021) and, however, it negatively affects the agar gelation and strength that properties mainly determine the target applications of agar products (Armisen, 1995). The total sulfate content and distinct features of organic and inorganic sulfate contents of water‐ and NaOH‐pretreated agars were analyzed using standard calibration equation of sulfate ions. The present study found significantly (*p* < .05) higher sulfate content in water‐pretreated agar (3.14% in dry mass) than in NaOH‐pretreated agar (1.27% in dry mass; Figure 4). Moreover, organic and inorganic sulfate contents of water‐ and NaOH‐pretreated agars were recorded as 2.739 and 0.26% (in dry mass), and 0.395 and 1.0% (in dry mass), respectively. A higher level of organic and inorganic sulfate contents was reported in study conducted on *G. domingensis* as 6.2 and 1.4% in dry mass, respectively (Torres et al., 2021). The alkali treatment decreased the sulfate content in agar and forming higher amounts of the gel‐inducing 3,6‐anhydrogalactose units (Rhein‐Knudsen et al., 2017). Similar studies of negative correlation between sulfate contents and gel strength properties were reported by Villanueva et al. (2010) and Martínez‐Sanz et al. (2019). The present findings suggest that NaOH‐pretreated agar from *G. tenuistipitata* could be suitable for food applications with moderate gel strength and considerable amount of biofunctional sulfated polysaccharides obtained.

#### 3.4.2 FTIR value of water‐ and NaOH‐pretreated agar

**FIGURE 5:**
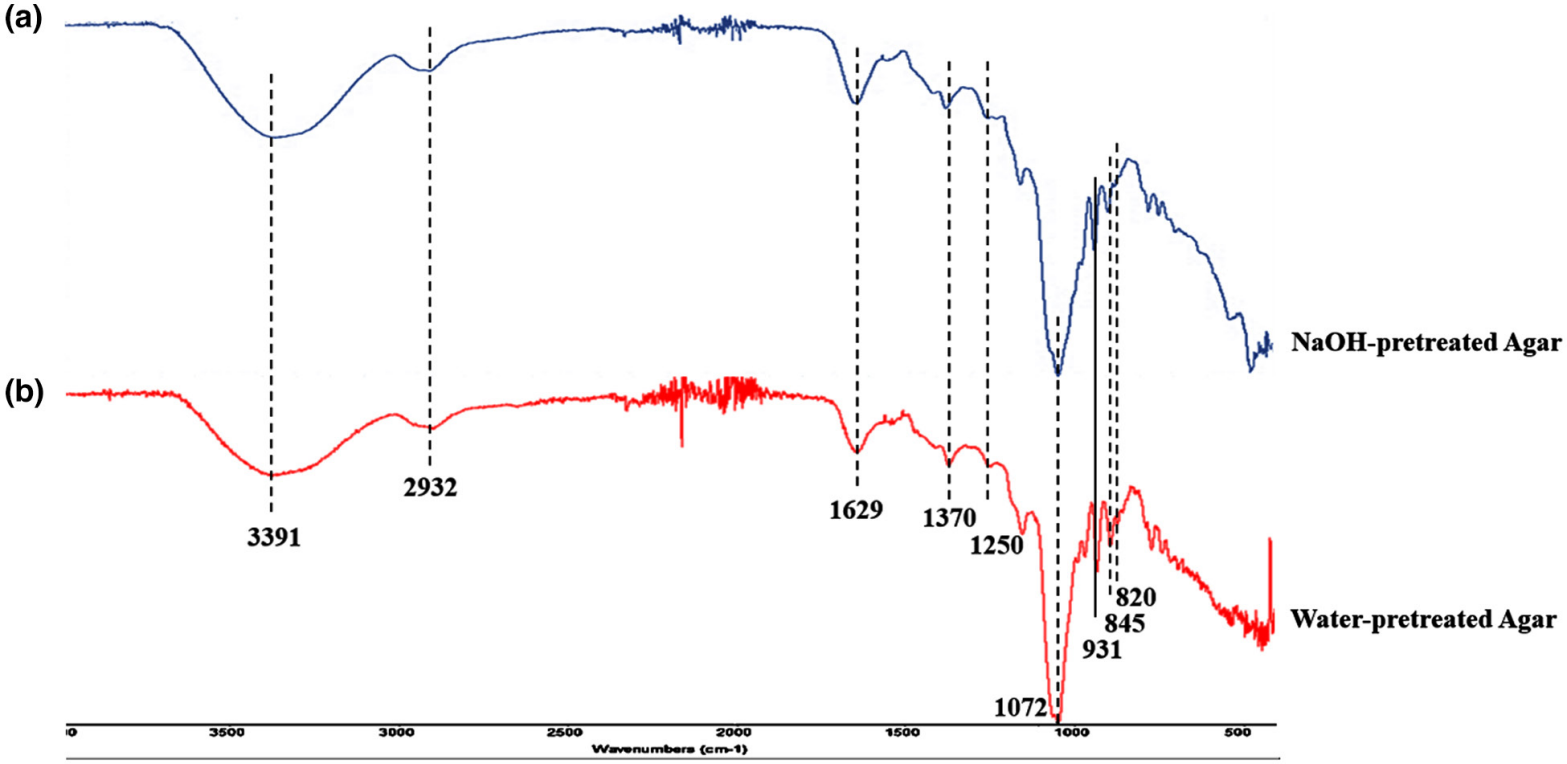
FTIR spectrum of water (a)‐ and NaOH (b)‐pretreated agars.

Fourier transform infrared spectra were analyzed to identify the major compositional differences of agar extracted from both water and NaOH pretreatment. The FTIR spectra bands were presented for both extracted agar in range of 3500 to 500 cm^−1^ (Figure 5). The major bands of 820, 845, 931, 1072, 1250, 1370, 1629, 2932, and 3391 cm^−1^ were observed for both water and NaOH‐pretreated agar (Figure 5). The presence of 845 cm^−1^, 820 cm^−1^, and 931 cm^−1^ suggests the existence of sulfate groups at the C‐4 position in the D‐galactose units, C‐6 of L‐galactose units and 3,6‐anhydro‐D‐galactose C‐O bonds (Gómez‐Ordóñez & Rupérez, 2011; Souza et al., 2012). Another study found *G. tenuistipitata* strong pick at 1026.3 and 1067.5 cm^−1^ is due to the C–O stretch of primary alcohol (Sobuj et al., 2021). The high amount of glucose detected in the agar bands between 990 and 1150 cm^−1^ (Rhein‐Knudsen et al., 2017). It was evident that most of the agar characteristic bands were located at approximately the same wave number, differences in relative intensities, especially when comparing the nonpurified agar extracts to the purified ones (Martínez‐Sanz et al., 2019). The present study was in line with the study of Rhein‐Knudsen et al. (2017) and Martínez‐Sanz et al. (2019) as they reported the relative intensity of these spectra was stronger and visible for alkaline‐pretreated agar, suggesting that higher degree of purity was attained. It is worth mentioning that the relative band intensity of the 3,6‐anhydrogalactose residue to that of the 6‐sulfate on the L‐galactose unit was higher in NaOH‐pretreated agar than that of water‐pretreated one, confirming the higher degree of conversion of L‐galactose 6‐sulfate to 3,6‐anhydrogalactose, due to alkali pretreatment in the present study.

#### 3.4.3 Chlorophylls and carotenoid content in water‐ and NaOH‐pretreated agar

**FIGURE 6:**
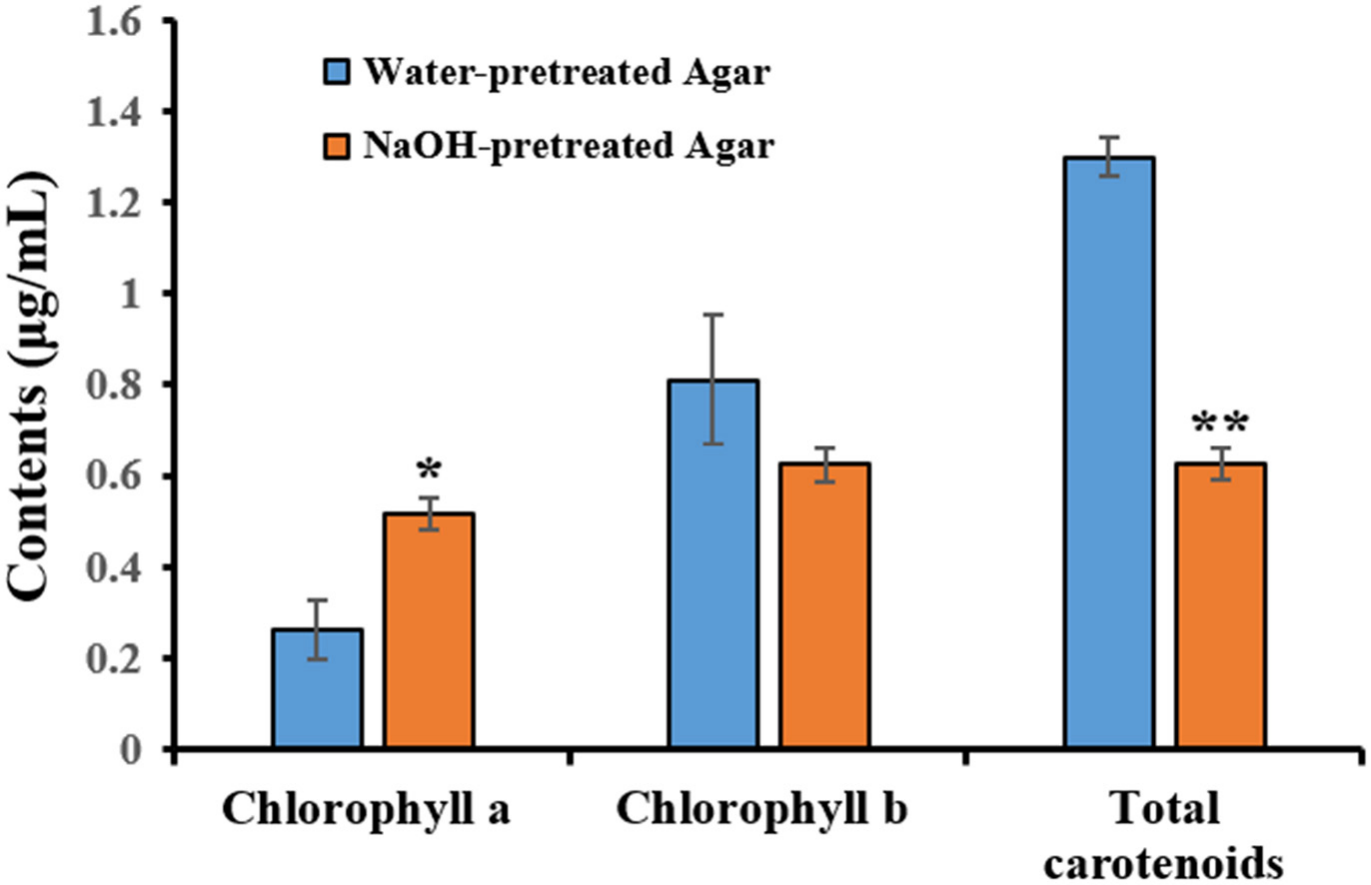
Chlorophyll (a and b) and total carotenoids in water‐ and NaOH‐pretreated agars. Data were presented as mean ± SE (*n* = 3), statistically significant at 95% and 99% confidence levels (*p < .05 and **p < .01).

Chlorophylls (a and b) and total carotenoids content were significantly higher in water‐pretreated agar (0.26, 0.81, and 1.3 μg/mL, respectively) than NaOH‐pretreated agar (0.52, 0.62 and 0.63 μg/mL, respectively; Figure 6). It was reported that pigments such as chlorophyll, carotenoid, and phycoerythrobilin were leached out in solution during alkali pretreatment, resulting from algal photolysis (Li et al., 2008; Xiao et al., 2021). The previous study investigated the similar chlorophyll and carotenoids contents in raw *G. tenuistipitata* seaweed (Hossain et al., 2021), where the results were higher than those of our present study, because of this study analyzed on water‐ and NaOH‐ pretreated agar. Scientific evidence from in vitro and in vivo studies shows that algal carotenoids are powerful natural antioxidants and are attributed to potential pharmacological uses in human health and disease management. (Mohibbullah et al., 2018, 2022). Therefore, considering higher gel strength and coloration, alkali‐pretreated agar containing natural pigments could be a potential source for antioxidants upon consumption as food materials.

#### 3.4.4 Antioxidative activity of water‐ and NaOH‐pretreated agar

**FIGURE 7:**
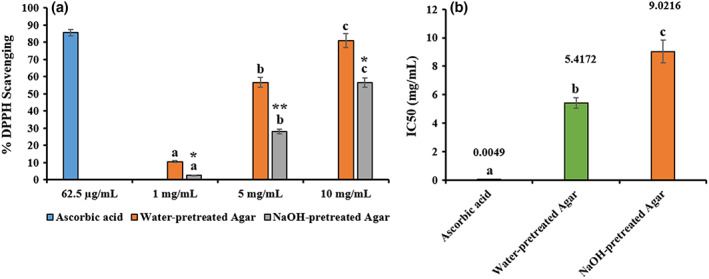
Antioxidant activity at different concentrations of water‐ and NaOH‐ pretreated agars (a). IC50 values of ascorbic acid, water‐ and NaOH‐pretreated agars (b). Data were presented as mean ± SE (*n* = 3), different letters in superscript showing the statistically significant at 95% confidence level (*p < .05).

In DPPH scavenging assay, nitrogen free radicals in the DPPH are easily scavenged by the potential antioxidants, and the purple color of the DPPH solution is eliminated by the antioxidants. Following this mechanism, water‐ and NaOH‐pretreated agar extracts significantly (*p* < .05) increased the antioxidant activity as the concentrations increase and the results were comparable with ascorbic acid as positive control (Figure 7a). The percentage of DPPH inhibition was higher in water‐pretreated agar than NaOH‐pretreated ones. Moreover, compared with ascorbic acid (IC50 = 0.0049 mg/mL), IC50 value of both agar extracts exhibited lower DPPH scavenging activity (Figure 7b). These antioxidant activities shown by both agar extracts were attributed to the presence of various phytochemicals especially algal pigments like carotenoids, as evidenced by the present study. It has been reported that *G. tenuistipitata* shows promising antioxidant activities in vitro (Sobuj et al., 2021). Similar trend of DPPH scavenging activity was shown in agar from brown seaweed and found to be elevated level (83.70 ± 3.83%; *p* < .05; Reshma et al., 2022). Therefore, it is concluded that agar extracted from *G. tenuistipitata* has a great potential for functional food applications.

## 4 CONCLUSIONS

Developmental optimization of different extraction variables, physicochemical properties, and antioxidant characterizations of food grade agar extracted from *Gracilaria tenuistipitata* of Bangladesh coast has reported, so far, for the first time. In this study, agar yield with better physicochemical properties was critically investigated in both water and NaOH pretreatment conditions for sustainable utilization of underutilized seaweed resources in Bangladesh. Results suggested that 2% NaOH pretreatment at 30°C for 3 h and seaweed to water ratio at 1:150, followed by extraction temperature at 100°C for 2 h, were shown to have food grade quality agar that required by the industry. Gel strength is a critical parameter for extracted agar and, in the present study, alkali pretreatment improved the gel strength in the range of food applications, although the value was far lower than commercial agar. Except of this, other physical and chemical parameters were comparable with commercial agar. Alkali pretreatment reduced the sulfate contents with other impurities like proteins, polyphenols, and carotenoids caused dark brown coloration, as confirmed by FTIR analysis, resulting in decreased antioxidant activity than water‐pretreated agar but still preserving antioxidant potentials at moderate level. Therefore, our study might be helpful to the local processors to introduce quality agar products as biofunctional food items by following the low‐cost extraction technology with improved extraction yield.

## CONFLICT OF INTEREST STATEMENT

No conflict of interest.

## Data Availability

Data will be made available on request.

## References

[fsn33265-bib-0001] Agriculture Organization of the United Nations. Fisheries, D . (2000). The state of world fisheries and aquaculture, 2000 (Vol. 3). Food & Agriculture Org.

[fsn33265-bib-0002] Alehosseini, A. , del Pulgar, E. M. G. , Gómez‐Mascaraque, L. G. , Martínez‐Sanz, M. , Fabra, M. J. , Sanz, Y. , & Lopez‐Rubio, A. (2018). Unpurified Gelidium‐extracted carbohydrate‐rich fractions improve probiotic protection during storage. LWT, 96, 694–703. 10.1016/j.lwt.2018.06.043

[fsn33265-bib-0003] Armisen, R. (1995). World‐wide use and importance of *Gracilaria* . Journal of Applied Phycology, 7(3), 231–243. 10.1007/BF00003998

[fsn33265-bib-0004] Arvizu‐Higuera, D. L. , Rodriguez‐Montesinos, Y. E. , Murillo‐Alvarez, J. I. , Munoz‐Ochoa, M. , & Hernandez‐Carmona, G. (2008). Effect of alkali treatment time and extraction time on agar from *Gracilaria vermiculophylla* . Journal of Applied Phycology, 20, 515–519. 10.1007/sl08lI-007-9258

[fsn33265-bib-0005] Belattmania, Z. , Bentiss, F. , Jama, C. , Nadri, A. , Reani, A. , & Sabour, B. (2021). Spectroscopic characterization and gel properties of agar from two *Gelidium* species from the Atlantic coast of Morocco. Biointerface Research in Applied Chemistry, 11, 12642–12652. 10.33263/BRIAC115.1264212652

[fsn33265-bib-0006] Brasch, D. J. , Chuah, C. , & Melton, L. D. (1984). The commercial agar from New Zealand Pterocladia species. Australian Journal of Chemistry, 37(1), 183–190. 10.1071/CH9840183

[fsn33265-bib-0007] Chen, H. M. , Zheng, L. , & Yan, X. J. (2005). The preparation and bioactivity research of Agaro‐oligosaccharides. Food Technology and Biotechnology, 43(1), 29–36.

[fsn33265-bib-0008] Durairatnam, M. (1987). Studies of the yield of agar, gel strength and quality of agar of *Gracilaria edulis* (Gmel.) Silva from Brazil. In M. A. Ragan , & C. J. Bird (Eds.), Twelfth International Seaweed Symposium. Developments in Hydrobiology (Vol. 41, pp. 509–512). Springer. 10.1007/978-94-009-4057-4_75

[fsn33265-bib-0009] Freile‐Pelegrın, Y. , & Murano, E. (2005). Agars from three species of *Gracilaria* (Rhodophyta) from Yucatán peninsula. Bioresource Technology, 96(3), 295–302. 10.1016/j.biortech.2004.04.010 15474929

[fsn33265-bib-0010] Freile‐Pelegrin, Y. , Robledo, D. R. , & García‐Reina, G. (1995). Seasonal agar yield and quality in *Gelidium canariensis* (Grunow) Seoane‐Camba (Gelidiales, Rhodophyta) from gran Canaria, Spain. Journal of Applied Phycology, 7(2), 141–144. 10.1007/BF00693060

[fsn33265-bib-0011] Gioele, C. , Marilena, S. , Valbona, A. , Nunziacarla, S. , Andrea, S. , & Antonio, M. (2017). *Gracilaria gracilis*, source of agar: A short review. Current Organic Chemistry, 21(5), 380–386.

[fsn33265-bib-0013] Gómez‐Ordóñez, E. , & Rupérez, P. (2011). FTIR‐ATR spectroscopy as a tool for polysaccharide identification in edible brown and red seaweeds. Food Hydrocolloids, 25(6), 1514–1520. 10.1016/j.foodhyd.2011.02.009

[fsn33265-bib-0014] Guerrero, P. , Etxabide, A. , Leceta, I. , Peñalba, M. , & De la Caba, K. (2014). Extraction of agar from *Gelidium sesquipedale* (Rodhopyta) and surface characterization of agar based films. Carbohydrate Polymers, 99, 491–498. 10.1016/j.carbpol.2013.08.049 24274535

[fsn33265-bib-0015] Hossain, M. T. , Sohag, A. A. M. , Haque, M. N. , Tahjib‐Ul‐Arif, M. , Dash, R. , Chowdhury, M. T. H. , & Hannan, M. A. (2021). Nutritional value, phytochemical profile, antioxidant property and agar yielding potential of macroalgae from coasts of Cox's Bazar and St. Martin's Island of Bangladesh. Journal of Aquatic Food Product Technology, 30(2), 217–227. 10.1080/10498850.2020.1869876

[fsn33265-bib-0016] Hurtado‐Ponce, A. , & Umezaki, I. (1988). Physical properties of agar gel from *Gracilaria* (Rhodophyta) of The Philippines. Botanica Marina, 31(2), 171–174. 10.1515/botm.1988.31.2.171

[fsn33265-bib-0017] Israel, A. , Martinez‐Goss, M. , & Friedlander, M. (1999). Effect of salinity and pH on growth and agar yield of *Gracilaria tenuistipitata* var. liui in laboratory and outdoor cultivation. Journal of Applied Phycology, 11(6), 543–549. 10.1023/A:1008141906299

[fsn33265-bib-0018] Istini, S. , Ohno, M. , & Kusunose, H. (1994). Methods of analysis for agar, carrageenan and alginate in seaweed. Bulletin of Marine Sciences and Fisheries, 14, 49–55.

[fsn33265-bib-0019] Joint, F. A. O. W. H. O. E. C. o. F. A. M. , & World Health Organization . (2007). Evaluation of certain food additives and contaminants: Sixty‐eighth report of the Joint FAO/WHO expert committee on food additives (Vol. 68). World Health Organization.

[fsn33265-bib-0020] Kumar, V. , & Fotedar, R. (2009). Agar extraction process for *Gracilaria cliftonii* (Withell, Millar, & Kraft, 1994). Carbohydrate Polymers, 78, 813–819. 10.1016/j.carbpol.2009.07.001

[fsn33265-bib-0021] Lee, W. K. , Namasivayam, P. , & Ho, C. L. (2014). Effects of sulfate starvation on agar polysaccharides of *Gracilaria* species (Gracilariaceae, Rhodophyta) from Morib, Malaysia. Journal of Applied Phycology, 26(4), 1791–1799. 10.1007/s10811-013-0231-0

[fsn33265-bib-0022] Li, H. , Yu, X. , Jin, Y. , Zhang, W. , & Liu, Y. (2008). Development of an eco‐friendly agar extraction technique from the red seaweed *Gracilaria lemaneiformis* . Bioresource Technology, 99(8), 3301–3305. 10.1016/j.biortech.2007.07.002 17765536

[fsn33265-bib-0023] Martínez‐Sanz, M. , Gómez‐Mascaraque, L. G. , Ballester, A. R. , Martínez‐Abad, A. , Brodkorb, A. , & López‐Rubio, A. (2019). Production of unpurified agar‐based extracts from red seaweed *Gelidium sesquipedale* by means of simplified extraction protocols. Algal Research, 38, 101420. 10.1016/j.algal.2019.101420

[fsn33265-bib-0024] McHugh, D. J. (2003). A guide to the seaweed industry. Food and Agriculture Organization of the United Nations FAO Fisheries Technical Paper 441.

[fsn33265-bib-0025] Meena, R. , Prasad, K. , Ganesan, M. , & Siddhanta, A. K. (2008). Superior quality agar from *Gracilaria* species (Gracilariales, Rhodophyta) collected from the Gulf of Mannar, India. Journal of Applied Phycology, 20(4), 397–402. 10.1007/s10811-007-9272-6

[fsn33265-bib-0026] Mohibbullah, M. , Haque, M. , Khan, M. N. A. , Park, I. S. , Moon, I. S. , & Hong, Y. K. (2018). Neuroprotective effects of fucoxanthin and its derivative fucoxanthinol from the phaeophyte *Undaria pinnatifida* attenuate oxidative stress in hippocampal neurons. Journal of Applied Phycology, 30(6), 3243–3252. 10.1007/s10811-018-1458-6

[fsn33265-bib-0027] Mohibbullah, M. , Haque, M. N. , Sohag, A. A. M. , Hossain, M. T. , Zahan, M. S. , Uddin, M. J. , & Choi, J. S. (2022). A systematic review on marine algae‐derived Fucoxanthin: An update of pharmacological insights. Marine Drugs, 20(5), 279. 10.3390/md20050279 35621930 PMC9146768

[fsn33265-bib-0028] Öğretmen, Ö. Y. , & Kaya, Y. (2019). Seasonal changes in the yield and gel properties of agar extracted from *Gelidium latifolium* (Rhodophyta). Journal of Applied Phycology, 31(5), 3091–3100. 10.1007/s10811-019-01786-w

[fsn33265-bib-0029] Prasad, K. , Siddhanta, A. K. , Ganesan, M. , Ramavat, B. K. , Jha, B. , & Ghosh, P. K. (2007). Agars of *Gelidiella acerosa* of west and southeast coasts of India. Bioresource Technology, 98(10), 1907–1915. 10.1016/j.biortech.2006.07.028 16949817

[fsn33265-bib-0030] Reshma, B. S. , Aavula, T. , Narasimman, V. , Ramachandran, S. , Essa, M. M. , & Qoronfleh, M. W. (2022). Antioxidant and antiaging properties of agar obtained from brown seaweed *Laminaria digitata* (Hudson) in D‐galactose‐induced swiss albino mice. Evidence‐Based Complementary and Alternative Medicine, 2022, 7736378. 10.1155/2022/7736378 35251211 PMC8894001

[fsn33265-bib-0031] Rhein‐Knudsen, N. , Ale, M. T. , Ajalloueian, F. , Yu, L. , & Meyer, A. S. (2017). Rheological properties of agar and carrageenan from Ghanaian red seaweeds. Food Hydrocolloids, 63, 50–58. 10.1016/j.foodhyd.2016.08.023

[fsn33265-bib-0032] Sasuga, K. , Yamanashi, T. , Nakayama, S. , Ono, S. , & Mikami, K. (2017). Optimization of yield and quality of agar polysaccharide isolated from the marine red macroalga *Pyropia yezoensis* . Algal Research, 26, 123–130. 10.1016/j.algal.2017.07.010

[fsn33265-bib-0033] Sobuj, M. K. A. , Islam, M. , Mahmud, Y. , & Rafiquzzaman, S. M. (2021). Effect of solvents on bioactive compounds and antioxidant activity of *Padina tetrastromatica* and *Gracilaria tenuistipitata* seaweeds collected from Bangladesh. Scientific Reports, 11(1), 1–13. 10.1038/s41598-021-98461-3 34580350 PMC8476583

[fsn33265-bib-0034] Sousa, A. M. , Morais, S. , Abreu, M. H. , Pereira, R. , Sousa‐Pinto, I. , Cabrita, E. J. , & Gonçalves, M. P. (2012). Structural, physical, and chemical modifications induced by microwave heating on native agar‐like galactans. Journal of Agricultural and Food Chemistry, 60(19), 4977–4985. 10.1021/jf2053542 22540146

[fsn33265-bib-0035] Sousa, A. M. , Souza, H. K. , Uknalis, J. , Liu, S. C. , Goncalves, M. P. , & Liu, L. (2015). Electrospinning of agar/PVA aqueous solutions and its relation with rheological properties. Carbohydrate Polymers, 115, 348–355. 10.1016/j.carbpol.2014.08.074 25439904

[fsn33265-bib-0036] Souza, B. W. , Cerqueira, M. A. , Bourbon, A. I. , Pinheiro, A. C. , Martins, J. T. , Teixeira, J. A. , & Vicente, A. A. (2012). Chemical characterization and antioxidant activity of sulfated polysaccharide from the red seaweed *Gracilaria birdiae* . Food Hydrocolloids, 27(2), 287–292. 10.1016/j.foodhyd.2011.10.005

[fsn33265-bib-0037] Torres, P. B. , Nagai, A. , Jara, C. E. P. , Santos, J. P. , Chow, F. , & Santos, D. Y. A. C. D. (2021). Determination of sulfate in algal polysaccharide samples: A step‐by‐step protocol using microplate reader. Ocean and Coastal Research, 69, 21021. 10.1590/2675-2824069.21-010pbt

[fsn33265-bib-0038] Truus, K. , Tuvikene, R. , Vaher, M. , Kailas, T. , Toomik, P. , & Pehk, T. (2006). Structural and compositional characteristics of gelling galactan from the red alga *Ahnfeltia tobuchiensis* (Ahnfeltiales, the sea of Japan). Carbohydrate Polymers, 63(1), 130–135. 10.1016/j.carbpol.2005.08.029

[fsn33265-bib-0039] Venugopal, V. (2016). Marine polysaccharides: Food applications. CRC Press.

[fsn33265-bib-0040] Villanueva, R. D. , Sousa, A. M. M. , Gonçalves, M. P. , Nilsson, M. , & Hilliou, L. (2010). Production and properties of agar from the invasive marine alga, *Gracilaria vermiculophylla* (Gracilariales, Rhodophyta). Journal of Applied Phycology, 22(2), 211–220. 10.1007/s10811-009-9444-7

[fsn33265-bib-0041] Wang, L. , Shen, Z. , Mu, H. , Lin, Y. , Zhang, J. , & Jiang, X. (2017). Impact of alkali pretreatment on yield, physico‐chemical and gelling properties of high quality agar from *Gracilaria tenuistipitata* . Food Hydrocolloids, 70, 356–362. 10.1016/j.foodhyd.2016.11.042

[fsn33265-bib-0042] Xiao, Q. , Wang, X. , Zhang, J. , Zhang, Y. , Chen, J. , Chen, F. , & Xiao, A. (2021). Pretreatment techniques and green extraction Technologies for Agar from *Gracilaria lemaneiformis* . Marine Drugs, 19(11), 617. 10.3390/md19110617 34822488 PMC8619328

[fsn33265-bib-0043] Yarnpakdee, S. , Benjakul, S. , & Kingwascharapong, P. (2015). Physico‐chemical and gel properties of agar from *Gracilaria tenuistipitata* from the lake of Songkhla, Thailand. Food Hydrocolloids, 51, 217–226. 10.1016/j.foodhyd.2015.05.004

[fsn33265-bib-0044] Yousefi, M. K. , Islami, H. R. , & Filizadeh, Y. (2013). Effect of extraction process on agar properties of *Gracilaria corticata* (Rhodophyta) collected from the Persian Gulf. Phycologia, 52(6), 481–487. 10.2216/13-165.1

[fsn33265-bib-0045] Zhao, P. , Wang, X. , Niu, J. , He, L. , Gu, W. , Xie, X. , & Wang, G. (2020). Agar extraction and purification of R‐phycoerythrin from *Gracilaria tenuistipitata*, and subsequent wastewater treatment by *Ulva prolifera* . Algal Research, 47, 101862. 10.1016/j.algal.2020.101862

